# Reply to Dablander et al.: Identifying interventions that change intentions lays a valuable foundation for behavior change

**DOI:** 10.1073/pnas.2513159122

**Published:** 2025-07-03

**Authors:** Alyssa H. Sinclair, Danielle Cosme, Kirsten Lydic, Diego A. Reinero, Michael E. Mann, Emily B. Falk

**Affiliations:** ^a^Penn Center for Science, Sustainability, and the Media, University of Pennsylvania, Philadelphia, PA 19104; ^b^Annenberg Public Policy Center, University of Pennsylvania, Philadelphia, PA 19104; ^c^Annenberg School for Communication, University of Pennsylvania, Philadelphia, PA 19104; ^d^Department of Psychology, University of Pennsylvania, Philadelphia, PA 19104; ^e^Department of Earth and Environmental Sciences, University of Pennsylvania, Philadelphia, PA 19104; ^f^Wharton Marketing Department, University of Pennsylvania, Philadelphia, PA 19104; ^g^Wharton Operations, Information and Decisions Department, University of Pennsylvania, Philadelphia, PA 19104

Dablander et al. highlight that intentions are distinct from behavior and propose that intervention studies must measure behavior to draw meaningful inferences (1). We appreciate the engagement with our work and call to action for the field. Our study focused on motivating action (i.e., increasing intentions); as stated in our paper, we did not measure behavior (2). We agree that changing behavior is a key goal of climate intervention research. However, it is not the only valid goal, and intentions are valuable predictors that can guide iterative research.

Meta-analyses indicate that intentions predict behavior, with moderately strong associations (r = 0.43 to 0.51) (3, 4). Crucially, stronger intentions are stronger predictors; across interventions, effect sizes for intentions are robustly associated with effect sizes for behavior (3, 4). In health behavior change, the gold standard for designing messages involves identifying changeable beliefs associated with intentions, which in turn predict longitudinal behavior change (5). This converging evidence demonstrates that intention strength is a meaningful signal. Although the magnitude of effects on behavior may be smaller, inferences about the relative effectiveness of interventions are supported by prior evidence.

Furthermore, the intention–behavior gap is smaller when perceived behavioral control is greater (3, 6). For this reason, we conducted formative research to identify actions that were rated highly on ease and capability to target in our tournament. Dablander et al. note—drawing partially on personal anecdotes—gaps between intentions and engagement in protests and civil disobedience. These actions are relatively difficult and pose risks of prosecution or injury, which could exacerbate the intention–behavior gap. The intention–behavior association is stronger for actions like reducing meat consumption (r = 0.53) (7). Our leading interventions increased intentions to engage in multiple individual and collective actions, suggesting broad potential despite the need to overcome action-specific barriers.

Dablander et al. highlight longitudinal studies (conducted by the last author) wherein a video intervention increased efficacy beliefs, but not real-world activism (8). However, this intervention did not demonstrate an intention–behavior gap as claimed; it did not increase intentions *or* behavior. Furthermore, these studies exemplify challenges in the field—changing behavior requires substantial time, effort, and funding. We systematically tested interventions to identify promising strategies to prioritize in future research. All interventions were theoretically grounded and had potential, but it would not be feasible or cost-effective to conduct this large-scale investigation with direct or longitudinal measures of all our target behaviors. Our tournament underscores the value of identifying promising interventions before investing resources in intensive studies of behavior.

Measuring intentions and behaviors can be complementary and iterative approaches. There is also value in outcomes beyond behavior; changing social norms, attitudes, and beliefs are important goals for addressing challenges like climate change that require collective action and policy change (9–11). In our tournament, we aimed to foreground promising interventions, generate insights into psychological mechanisms, and lay an empirical foundation to guide future research. In ongoing work, we are already striving to replicate our leading interventions with behavioral measures in longitudinal samples. We hope that others in the field will do the same.
